# Environmental Implication of Herbicide Use

**DOI:** 10.3390/molecules29245965

**Published:** 2024-12-18

**Authors:** Małgorzata Baćmaga, Jadwiga Wyszkowska, Jan Kucharski

**Affiliations:** Department of Soil Science and Microbiology, Faculty of Agriculture and Forestry, University of Warmia and Mazury in Olsztyn, Łódzki 3 Sq., 10–719 Olsztyn, Poland; jan.kucharski@uwm.edu.pl

**Keywords:** herbicides, contamination, environmental, toxicity, organisms, biodegradation

## Abstract

One of the guiding principles of the sustainable use of herbicides is their targeted action exclusively against weeds, consisting of blocking photosynthesis and synthesis of amino acids and growth regulators. Herbicides are major elements of plant production, indispensable to the functioning of modern agriculture. Nevertheless, their influence on all elements of the natural environment needs to be continuously controlled. This review article summarizes research addressing the effects of herbicides on the natural environment and the changes they trigger therein. Herbicides, applied to protect crops against weed infestation, are usually mixtures of various active substances; hence, it is generally difficult to analyze their impact on the environment and organisms. Nonetheless, an attempt was made in this review to discuss the effects of selected herbicides on individual elements of the natural environment (water, soil, and air) and organisms (humans, animals, plants, and microorganisms). In addition, the article presents examples of the biodegradation of selected herbicides and mechanisms of their degradation by bacteria and fungi. Based on this information, it can be concluded that the uncontrolled use of herbicides has led to adverse effects on non-target organisms, as documented in the scientific literature. However, further research on the environmental effects of these chemicals is needed address the missing knowledge on this subject.

## 1. Introduction

Herbicides represent a group of chemical substances extensively applied to protect crops from weeds in order to improve their yield quality and quantity [1]. Their use has been reported as early as in Greek and Roman times. Democritus was the first to describe a herbicide, i.e., lupine flowers soaked in hemlock juice. Highly saline water was also used to kill weeds. One of the first herbicides was a by–product of olive oil production called amurca, the use of which was described in the manuals of Cato the Elder in around 150 BC. The first effective chemical herbicide was 6% copper sulfate, used in the 1940s for selective control of mustard in cereal stands. In turn, the first commercial inorganic herbicide was sodium arsenate, and the first organic herbicide was sodium dinitrocresylate developed in 1896 in France [2,3]. Despite advantages stemming from herbicide use, the use of chemicals in agriculture has led to slow changes in many ecosystems. Most of these chemical compounds penetrate into the soil, a process which is severely hazardous to the natural environment. Their accumulation in the soil impedes plant production, diminishes food safety and, consequently, poses risk to human health [4]. Jara and Winter [5] have reported that only 1% of pesticides reach their targets, while their remaining part pervades the soil and undergoes multiple conversions. Their residues in the soil and their metabolites migrate to other ecosystems, most often to the atmospheric air, underground and surface waters, and bottom deposits. These chemicals may cause the loss of biodiversity in various ecosystems and accumulate in different organisms [6,7,8,9].

The occurrence and migration of herbicides in the environment is a matter of concern; hence, all of the factors contributing to the hazard posed to ecosystems and organisms exposed to herbicides should be considered during their registration [10]. Investigations addressing the behavior of herbicides in the natural environment enable a complete understanding of the dynamics of their migration across the environment, including in particular their retention, transport and conversion, which are strongly intertwined and usually affect one another. The behavior and migration of herbicides across the environment depend on the chemical composition of their active substances, the matrix and environmental conditions. Effective control of weed infestation in crop stands is feasible upon thorough understanding of the fate of herbicides in the environment, and especially the mechanisms of their interaction with the environment [11].

Physicochemical properties of herbicides are one of the key factors affecting their initial distribution and behavior in the environment. Thus, the behavior of herbicides from the same chemical group will be similar in a given environment. Their main physicochemical properties determining initial behavior in the environment include acid-base dissociation constant (pK_a_/pK_b_), solubility in water (S_w_), octanol–water partition coefficient (K_ow_), water vapor pressure (VP), sorption coefficient (K_d_ and K_oc_), half-life (DT_50_), and residual life in plants (RL_50_). These factors are particularly important in the case of pre-emergence herbicides applied at the onset of the growing season when both temperature and microbiological activity are relatively low [11,12,13,14]. During the application of herbicides, most of their active substances penetrate into the soil. Therefore, a perfect herbicide should have a half-life of less than 90 days, lipophilicity of log*P* < 2, and a recommended active substance dose below 200 g per 1 ha. The half-life of its active substance should not be too long, given the fact that it could cause potential contamination of underground waters, or problems associated with selectivity in successive crops. Therefore, in order for this active substance to migrate properly in the soil, it must have adequate solubility in water and mobility in the environment [14].

Taking into account all the above premises, this review article aimed to present (i) the effect of herbicides on the functioning of aquatic, soil and air ecosystems; (ii) migration of herbicides across the environment; (iii) outcomes of herbicide effects on organisms, i.e., humans, animals, plants, and microorganisms; and (iv) the potential of microorganisms for herbicide conversion in the environment, in the light of recent literature data. An overview of research into the interactions of herbicides with the environment and organisms, as well as their in-depth characterization, represents a viable approach in systematizing knowledge on their toxicity and on disturbances observed in the natural environment exposed to their action.

## 2. Importance and Characteristics of the Most Commonly Used Herbicide Classes

**Figure 1:**
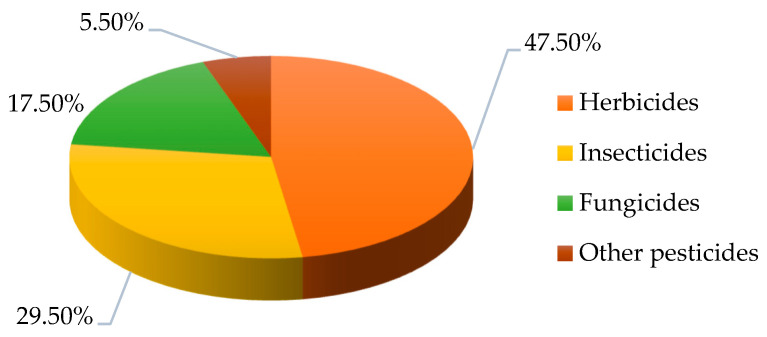
Percentage of use of each group of pesticides worldwide in 2022. Own elaboration based on Statista Research Department [16].

The global agricultural sector suffers from damage inflicted by 8000 weed species, with these losses equating to 13% of the entire crop harvest. Herbicides play a crucial role in safeguarding crops against weeds and ensuring robust agricultural yields, representing the predominant portion of pesticide usage worldwide [15], which stands at 47.5% (Figure 1).

Annual global herbicide consumption in 2020 was 1.397 million tons. Herbicide consumption by continent is as follows (from highest to lowest): South America (0.551 million tons), North America (0.315 million tons) and Central America (0.032 million tons). China, the United States, Argentina, Thailand, Brazil, Italy, France, Canada, Japan and India consumed most of these chemicals in 2020 [17,18]. The largest importers of herbicides from India are China, Japan and Israel. In contrast, the world’s largest suppliers of herbicides are the United States, Japan and China. The world’s leading consumers of pesticides are China (14.0 kg ha^−1^), Japan (11.0 kg ha^−1^) and the United States (4.5 kg ha^−1^), while the world average is 3.0 kg ha^−1^ [19].

**Figure 2:**
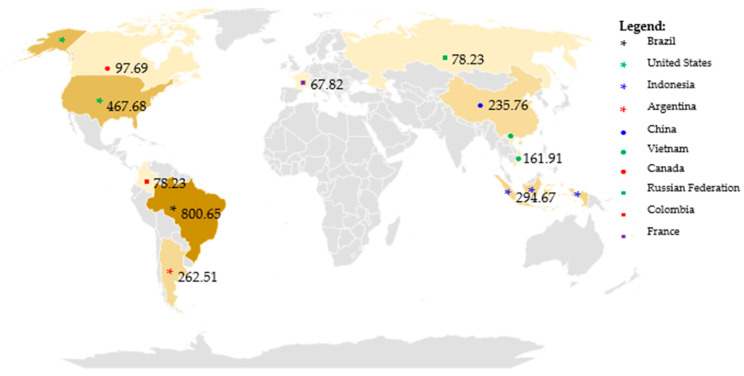
Countries which use the most pesticides in agriculture in 2022 (in 1.0 ton). Own elaboration based on Statista Research Department [16].

Figure 2 shows the 10 countries that use the largest amounts of pesticide per ton. These countries include Brazil, the United States, Indonesia, Argentina, China, Vietnam, Canada, Russian Federation, Colombia and France [16].

Herbicides are chemicals that are designed to destroy weeds while not damaging crop plants. They readily penetrate plants through the root system or leaves, as well as through the shoots. It has been noted that herbicides can be taken up by swelling seeds. The mechanism of action of herbicides on weeds involves the disruption of biochemical and biophysical processes in the plant, which are closely related to the metabolism and life processes of plant cells [20].

The most comprehensive classification system for herbicides developed by the Global Herbicide Resistance Action Committee (HRAC) is their division in terms of chemical properties and mechanism of action. The most commonly used herbicide groups for crop weed control include [21,22,23]:

1. Acetyl–CoA carboxylase (ACC) inhibitors—inhibit fatty acid synthesis and destroy cell membrane structure; aryloxy-phenoxy propionate group compounds.
2. Acetyl–lactate synthase (ALS) inhibitors—inhibit the production of the branched-chain amino acids leucine, valine and isoleucine; compounds from the cyclohexanedione, imidazolinone, pyrimidinylthio-benzoate, sulfonyl-amino-carbonyl-triazolinone and sulfonylurea group of compounds.
3. Inhibitors of the microtubule system—inhibit cell division in plant roots; triazolo-pyrimidine and dinitroaniline group compounds.
4. Synthetic auxins—inhibit cell growth in newly forming leaves and stem and the nucleic acid metabolism, and interfere with cell wall plasticity; compounds in the pyridine, phenoxy, benzoic acid, carboxylic acid, and quinaline carboxcylic acid groups.
5. Photosynthesis inhibitors at photosystem II level—cause blockage of electron transport in the second stable electron acceptor of photosystem II, which interrupts photosynthesis and energy production; compounds in the group phenyl-carbamate, pyridazinone, triazine, triazinone, triazolinone, uracil, benzothiadiazoles, nitriles, phenyl-pyridazine, amide, and urea.
6. Lipid synthesis inhibitors—inhibit the production of cuticular wax and inhibit shoot growth; compounds from the thiocarbamates group.
7. 5-enolpyruvylshikimate-3-phosphate synthase (EPSP) inhibitors—inhibit the synthesis of aromatic amino acids; compounds of the organophosphates group.
8. Glutamine synthetase inhibitors—cause accumulation of huge amounts of ammonia, so that plant cells are destroyed.
9. Carotenoid biosynthesis inhibitors/inhibitors of pigment synthesis—damage chlorophyll pigments; compounds from the triazoles, pyridazinone, and isoxazolidinnes group.
10. Cell membrane inhibitors (PPOs)—uses disruption of cell membrane function; compounds in the diphenyl-methyl ester group, bipyridylium, N-phenyl-phthalimides, and oxadiazoles.

The most commonly used chemical groups of herbicides in crop protection and their structural formulas are shown in Table 1. 

According to the Pesticide Properties Database (PPDB), herbicides also differ in their duration of action and persistence in the soil, with half-life in the soil ranging from 2 days to 300 days. Therefore, herbicides are divided into three groups [34]:

▪ Persistent—degrade 75%–100% from 2 to 3 years;
▪ Moderately persistent—degrade from 1 to 18 months;
▪ Non-persistent—degrade up to 12 weeks.

Herbicides are also divided according to their mode of action and weed control spectrum. There are two groups of herbicides: selective and non-selective herbicides. Selective herbicides control specific species of weeds, do not damage crop plants, and act on weeds through specific mechanisms that are effective against specific weeds. Examples of selective herbicides are acetochlor, alachlor, amidosulfuron, atrazine, benazolin, bentazone, bromoxynil, chlorpropham, clopyralid, clomazone, chlorosulfuron, chloro-toluron, 2,4-D, desmediphan, di-flufenican, dicamba, diuron, etofumesat, flufenacet, isoproturon, and mezotrion. Non-selective herbicides, on the other hand, are potent substances used mainly in areas overgrown with undesirable plants and have a very broad spectrum of action, eliminating most of the weeds they come into contact with. Non-selective herbicides include glyphosate, glufosinate, paraquat, and dichloride [14].

Most herbicides must be applied at the right time to achieve high efficacy in weed control and selectivity towards the crop [14,23]. Considering the timing of application in relation to the crop, herbicides are divided into three groups [14,25]:

▪ Pre-sowing (applied before sowing or planting a crop);
▪ Post-emergence (applied after the crop emerged);
▪ Pre-emergence (applied after the sowing of the crop and before its emergence).

The World Health Organisation (WHO) [15,35,36] divided herbicides into four groups, including two subgroups (Ia and Ib) for Group 1, taking into account their toxicity to non-target organisms (Table 2).

## 3. Sources and Residues of Herbicides in the Environment

The penetration and movement of herbicides in the environment are determined by multiple factors, but a very important influence is the addition of other chemicals [37]. Gandhi et al. [38] emphasize that herbicide toxicity is increased by the adjuvants used in their formulation, which interact with the herbicide’s active ingredients, and this may pose a greater risk to non-target organisms. Adjuvants primarily increase the efficacy of herbicides for weed control but can also contribute to the formation of persistent combinations in the environment, leading to side effects in plants, animals and humans [23]. De Castro et al. [39] report that prolonged and incompetent use of herbicides is often the cause of their accumulation in the environment, mainly in soil and aquatic ecosystems. Factors affecting the movement of herbicides in the environment include [23,40,41]

1. Herbicide persistence—associated with sorption and desorption processes in the environment, which can lead to the accumulation or removal of herbicides as a result of interactions of these compounds with plants, soil, water and sediments.
2. Mobility of herbicides, which is related to
  ▪ surface run-off—transport of herbicides due to rainfall and land irrigation;
  ▪ leaching—vertical movement deep into the soil profile caused by irrigation or rainfall;
  ▪ volatilization into the atmosphere—loss of herbicides through evaporation from water, soil and plants;
  ▪ translocation of herbicides—transport of herbicide droplets caused by wind action.
3. Herbicide degradation:
  ▪ chemical degradation—occurs during hydrolysis, redox reactions and ionization of herbicides;
  ▪ photodegradation—degradation of herbicides occurs when exposed to solar radiation;
  ▪ microbial degradation—transformation of herbicides by microorganisms, which is greatly influenced by the abundance, diversity and activity of microorganisms present in the environment.

By the time herbicides are applied to a specific area or plant, they already have the potential to migrate and degrade in the environment with the involvement of various biotic and abiotic factors. These chemicals degrade in the environment under the influence of microbial activity and various physicochemical factors, i.e., sunlight, temperature, humidity, and oxygen content. During these processes, they are transformed into metabolites that may be non-toxic or more harmful than the starting compound itself [42]. Herbicides and their metabolites move through the environment by adsorption, leaching, volatilization or surface run-off [43].

### 3.1. Water

Malaj et al. [44] and McGinley et al. [45] report that anthropogenic activities, including the use of herbicides, pose a huge threat to the integrity and biodiversity of water bodies. Herbicides for weed control can be directly applied to surface waters. The most common aquatic invasive species are *Phragmites australis*, *Hydrilla verticillata*, *Eichornia crassipes* and *Myriophyllum spicatum*, and herbicides such as glyphosate, 2,4-D, picloram, diquat and triclopyr are used to control them [46]. These chemicals can be transported to surface waters via multiple pathways, mainly through run-off and drainage from agricultural fields [47]. Direct over-spraying of trees in forests can be a source of herbicide infiltration into surface waters [48]. Urban wastewater treatment plants, storm sewers, and run-off from urban areas can also be sources of herbicide water pollution [49]. Zang [50] reports that herbicide run-off into ditches surrounding agricultural fields spreads to surface water from point and diffuse sources, and then flows to groundwater. Among the herbicide-active ingredients most commonly found in waters are amides, phenoxy hormone products, bipyridyls, urea derivatives, dinitroanilines, carbamates, sulfonylurea and uracil.

When herbicides enter surface waters, they are subject to dissolution, which can lead to their release in the water, resulting in high toxicity to free-living plants and phytoplankton [46,51].

The occurrence of herbicides in the aquatic environment is determined by a number of factors, among which are the chemical properties and formulation of the preparation, environmental conditions, the method of herbicide application, and the time and rate of dissemination in water [52]. The interaction of all the above-mentioned factors affects the retention, degradation and transport processes of herbicides in the aquatic environment. The ability of herbicides to penetrate and accumulate in water is also determined by the value of the octanol-water ratio (K_ow_). The higher the coefficient, the more hydrophobic the herbicides, making them accumulate in higher amounts in bottom sediments (sorption on organic matter particles and clay) than herbicides with lower K_ow_ [49,51].

Herbicides found in the aquatic environment may interact with organic matter, resulting in the absorption of active substances [51,52]. As a result of this process, aggregates are formed that sink to the bottom of water reservoirs and transport herbicides to bottom sediments, which are considered the main absorbents in water. Moreover, herbicides can be adsorbed or taken up by aquatic plants such as *Spirodela polyrhiza*, *Cabomba aquatica*, *Ludwigia peploides*, *Myriophyllum aquaticum*, *Elodea canadensis*, *Lemna minor* and *Eichhornia crassipes* [53]. The transformation of herbicides in water depends on pH, redox processes, access to light and temperature [54]. Water pH, by changing the herbicide load, can determine the rate of herbicide hydrolysis and degradation. Microorganisms play an important role in the decomposition of herbicides present in sludge and, under appropriate conditions, effectively degrade these compounds. When anaerobic conditions occur in water or aquatic sediments, the decomposition process of herbicides is generally slowed down [55]. With adequate access to oxygen near the roots of aquatic plants, the biodegradation process of these xenobiotics occurs intensively due to the rapid multiplication of microorganisms that use plant root secretions for their growth and development [56,57]. Mercurio et al. [54] and Vieirai et al. [58] report that the metabolites formed during the degradation of herbicides are generally less toxic than the starting compound itself because they are more polar and soluble in water.

In surface waters and ecosystem sediments, the most commonly detected herbicides are acetochlor, metazachlor, metolachlor, atrazine, bentazone, diuron, irgarol, isoproturon, simazine, symmetrine, terbuthylazine, terbutrin, flutramone, MCPA, and mecoprop [56,57].

An et al. [59] report that, within China (36 cities), out of 278 drinking water samples, acetochlor was detected in 189 samples at an average of 2.71 × 10^−8^ µg dm^−3^. A slightly lower content of acetochlor at an average of 2.66 × 10^−8^ µg dm^−3^ was detected in Dalian city in rivers and reservoirs. Jorfi et al. [60] report that high contents of acetochlor, metolachlor, syzamzine, metribuzine and atrazine ranging from 0.4 µg dm^−3^ to 13 µg dm^−3^ were found in the Tejo, Guadiana and Sado river basins in Portugal.

In surface waters in the Cachapoal River basin in Chile, desmethyl-terbuthylazine, pyrimethanil, cyprodinil and diazinon were the most common substances in the water samples analyzed, while simazine and the decomposition product desmethyl-terbuthylazine (DET) were found in water in the highest amounts. Simazine ranged from 1.227 µg dm^−3^ to 14.707 µg dm^−3^, while desmethyl-terbuthylazine ranged from 1.512 µg dm^−3^ to 21.897 µg dm^−3^ [61].

Álvarez Bayona et al. [62] showed that the glyphosate content in the waters of the Pamplonita and Zulia rivers in the Cúcuta metropolitan area was 2016 µg dm^−3^ and 204.5 µg dm^−3^, respectively.

In a semi-arid region of Argentina in the agricultural areas in Santiago del Estero, herbicide content was monitored in rainwater harvesting tanks, wells and dams. The most common herbicides detected in the waters were atrazine and metolachlor, while glyphosate and aminomethyl-phosphonic acid (AMPA) were present in the highest amounts. The maximum concentration of glyphosate was 35 µg dm^−3^, while that for AMPA was 1.90 µg dm^−3^ [63].

In South Africa, simazine, atrazine, metolachlor and terbuthylazine were detected in 12 water samples from rivers, backwaters/reservoirs and treated drinking water. The highest concentration in the water was that of simazine, whose average content in rivers was 1.82 mg dm^−3^, dams/reservoirs 0.12 mg dm^−3^ and treated drinking water 0.03 mg dm^−3^. In other waters, the content of detected herbicides varied on average in rivers and dams/reservoirs from 0.01 mg dm^−3^ (metolachlor) to 0.06 mg dm^−3^ (terbuthylazine), and in treated drinking water from 0.01 mg dm^−3^ (metolachlor) to 0.02 mg dm^−3^ (terbuthylazine) [64].

In the northeastern United States in the state of Arkansas in agricultural areas, frequently detected herbicides in water were clomazone, glyphosate, metolachlor, quinclorac, 2,4-D, dicamba, and propanil. The contents of clomazone, glyphosate, and quinclorac were higher in drainage ditches (contents were 0.80–67 µg dm^−3^, 0.50–6.2 µg dm^−3^, and 0.40–62 µg dm^−3^) than in water storage tank (contents were 0.80–6.0 µg dm^−3^, 0.50–4.1 µg dm^−3^, and 0.40–6.0 µg dm^−3^, respectively) [65].

Six herbicides (isoproturon, atrazine, 2-hydroxy atrazine, terbuthylazine, diuron, and metolachlor) were detected in Lake Taihu and were present at all water sampling points. The maximum concentration of herbicides detected in the lake was isoproturon at 847 ng dm^−3^, atrazine at 1726 ng dm^−3^, 2-hydroxyatrazine at 2680 ng dm^−3^, terbuthylazine at 1687 ng dm^−3^, diuron at 107.3 ng dm^−3^, and metholchlor at 316 ng dm^−3^. In turn, in the Jiuli River flowing into Lake Taihu, atrazine was detected at a concentration of 19.0—1190 ng dm^−3^, metolachlor at 94.3 ng dm^−3^, nitrapyrin at 25.7 ng dm^−3^ and acetochlor at 26.0 ng dm^−3^ [66].

Glyphosate, which is excessively and widely used around the world to protect plants, poses a very serious threat to the aquatic environment. Glyphosate residues in water have been detected in European countries (Germany, France, Denmark, Spain, Hungary, Switzerland), Canada, the USA, Mexico and Argentina [67]. In South Africa, simazine was detected in water sources around Mangaung Metropolitan Municipality at an amount of 1.82 mg dm^−3^ in the river, 0.12 mg dm^−3^ in dams and 0.03 mg dm^−3^ in treated water, which constituted a high ecological risk in terms of acute and chronic toxicity [68]. Herbicides were also detected in drinking water. An example is glyphosate, which was present in drinking water in Australia and the United States in amounts of 1000 µg dm^−3^ and 700 µg dm^−3^, respectively [69].

The content of triazine herbicides in marine waters at 661 stations, 7 marine areas and 7 continents was analyzed by Yang et al. [70]. The highest concentrations of these herbicides were found in the waters of the Gulf of Mexico, East Asia and the Gulf of Vilaine, with average contents of 13.67 nmol dm^−3^, 12.07 nmol dm^−3^ and 11.68 nmol dm^−3^, respectively, and highest maximum concentrations of these herbicides at 3.84 nmol dm^−3^, 2.28 nmol dm^−3^ and 1.64 nmol dm^−3^, respectively.

### 3.2. Soil

The increase in the use of herbicides is due to the increasing area under cultivation and the development and spread of weed resistance, and this consequently leads to the use of higher doses of these compounds or the introduction of new active substances and formulations [71]. Soil is the most vulnerable environment for herbicide pollution, being an integral part of the natural environment, which includes interactions between organisms, air, water and other elements of the ecosystem. Due to its complexity, it is a huge storehouse that accumulates both nutrients necessary for the functioning of organisms and toxic compounds that disrupt all chemical and biochemical processes. Herbicides entering the soil can directly or indirectly affect its living organisms [22]. Herbicides are among the main contaminants most frequently detected in the soil environment, along with heavy metals or polycyclic aromatic hydrocarbons. The widespread use of herbicides is of growing concern because of the dangers they can cause to the environment. Improper use of herbicides or inadequate disposal of unused active ingredients can lead to bioaccumulation and residues in the soil, resulting in adverse environmental effects. According to Łozowicka et al. [72], there is never just one active substance of an agent in the soil, but a mixture of various substances that act in an additive, synergistic or antagonistic manner.

Vertical transport of herbicides takes place in the soil profile, i.e., the phenomenon of herbicide leaching, which is essential for the incorporation of active substances applied before crop emergence to destroy weeds in crops. The intensity of herbicide leaching into the soil depends on the method of application, the prevailing environmental conditions and the properties of the herbicide itself. The interactions between the aforementioned factors have a direct effect on the degradation of the herbicide in the soil profile and, consequently, on its effectiveness in controlling weeds and on potential environmental contamination [73].

Application of herbicide formulations in the form of a mixture of active substances is more effective in controlling weeds; however, their sorption in the soil is reduced. An example of this is the study by Silva et al. [74], which showed reduced sorption of sulfometron-methyl, diuron and hexazinone when they were applied in a mixture. This form of herbicide application led to a high loss of these active substances in the soil profile, and thus increased the risk of surface and groundwater contamination by these chemicals.

Herbicides present in the soil can penetrate other links of the post-food chain, e.g., to plants, animals and humans. The behaviour of herbicides in the soil environment depends on many factors, i.e., climatic factors, physical, chemical and biological properties of the soil, and the properties of the active substances that determine the persistence of herbicides in the environment (the form of the herbicide and its dose, degradation time, solubility and reactivity) [75].

Carles et al. [76] noted that metabolites of some herbicides, e.g., metazachlor, glycoside or diclofop, are more toxic than the starting compound itself. However, they stress that, in general, these substances are present in the soil in trace amounts, and therefore do not increase the mortality of the organisms present. At the same time, intensive and repeated application of these substances can lead to their accumulation in the soil, so they can become dangerous to non-target organisms by causing sub-chronic, chronic and subacute toxicity [77].

A huge danger in the soil environment is posed by mixtures of herbicide active substances and their interactions, which can lead to antagonism and synergy [22]. Soil physicochemical parameters under the influence of herbicides change slowly, and the impact of herbicide use or soil contamination is observed in the long-term use of these chemicals. Herbicides that penetrate the soil environment can affect the organisms in it in different ways. For example, diclofop and haloxyfop (ACC inhibitors) inhibit the proliferation of sulfur bacteria and the growth of rice. Other herbicides, i.e., sulfonylureas, imidazolinones, triazolo-pyrimidines, and glyphosate cause changes in the structure and functioning of soil microorganisms and lead to disorders in the growth of *Arabidopsis* sp. Herbicides diquat, paraquat, metolachlor, acetochlor, and pendimethalin reduce the growth of nitrogen-fixing bacteria and plants, e.g., *Oryza sativa* and *Triticum aestivum* L. In turn, triazine herbicides lead to modifications in the structure of microbial assemblages and the growth and development of *Millet Pennisetum americanum* L. [78].

Frequent use of herbicides reduces the population of microorganisms in the soil, which leads to an ecological imbalance [19]. The authors give examples of herbicides that contribute to changes in the soil microbiome. For example, atrazine at 3.4 kg ha^−1^ caused a temporary inhibition of bacterial growth, and at a dose of 10 µg dm^−3^ caused the complete disappearance of Cyanobacteria. Acetochlor at a dose of 1.25 to 5.0 dm^3^ ha^−1^ reduced the population of Actinobacteria and fungi. The presence of nicosulfuron in the soil at 0.3–15.0 mg kg^−1^ and metribuzin at 12.0–600 mg kg^−1^ reduced the population of Actinobacteria. Ferreira et al. [17] analyzed the occurrence of glyphosate and amino-methyl-phosphonic acid (AMPA) in soils around the world. The content of glyphosate in soils was detected at levels ranging from 2.5 × 10^−4^ to 66.38 mg kg^−1^ soil, while that of amino-methyl-phosphonic acid ranged from 2.5 × 10^−3^ to 38.94 mg kg^−1^ soil. Glyphosate was found to be present in 73% and amino-methyl-phosphonic acid in 68% of soils worldwide, while amino-methyl-phosphonic acid was found to be present in up to 1.0 mg kg^−1^ soil. There were two areas in North America, four areas in Europe, and one area in South America and Asia where glyphosate content ranged from 1.0 to 20 mg kg^−1^ soil. Glyphosate content of more than 20.0 mg kg^−1^ soil was recorded in South America (three areas) and Europe (one area). In the case of amino-methyl-phosphonic acid, a content of between 1.0 mg kg^−1^ and 20.0 mg kg^−1^ soil was found in five areas in South America and Europe, and of above 20.0 mg kg^−1^ soil in two areas in South America.

A study by Wang et al. [79] in an agricultural area in northeastern China showed that fomesafen, chlomazone, acetochlor, atrazine and metolachlor were the most commonly detected herbicides in soil. The concentrations of these herbicides in the soil were 3.0–391 μg kg^−1^, 1.0–218 μg kg^−1^, 0.0–133 μg kg^−1^, 1.0–48 μg kg^−1^ and 0.0–75 μg kg^−1^, respectively. The authors also observed that the long-term presence of herbicide residues significantly affects soil multifunctionality and microbial diversity. When the amount of herbicide analyzed exceeded 310 µg kg^−1^, the soil organic and total nitrogen content decreased. In addition, the diversity of soil microorganisms was also reduced.

In an agricultural area in the province of Vojvodina, Serbia, the occurrence of the most commonly used herbicides was determined in 41 soil samples in 128 localities. Herbicide monitoring was conducted in 2013 and 2023. In 2013, the most frequently detected herbicides in soil samples were 2.4-D-methyl ester (5.65–7.73 mg kg^−1^), bentazone (5.27–5.68 mg kg^−1^), napropamide (2.21–3.17 mg kg^−1^), metazachlor (1.29–1.44 mg kg^−1^), prosulfocarb (2.93–5.58 mg kg^−1^) and flumioxazin (0.86–1.83 mg kg^−1^). On the other hand, the following herbicides were detected in 2023 soil samples: aclonifene (6.83–9.42 mg kg^−1^), bentazone (7.70–9.02 mg kg^−1^), flumioxazin (0.72–1.10 mg kg^−1^), metazachlor (0.67–1.16 mg kg^−1^), napropamide (2.72–2.74 mg kg^−1^), propyzamide (2.34–3.47 mg kg^−1^), and prosulfocarb (5.86–5.97 mg kg^−1^) [80].

In Australia, soil samples were taken from cereal fields before sowing winter crops in 2015 and 2016 to determine herbicide residues [81]. The most commonly detected herbicide residues were glyphosate and amino-methyl-phosphonic acid (AMPA). The mean concentration of glyphosate in 2015 was 0.12 mg kg^−1^ and AMPA was 0.41 mg kg^−1^. In contrast, in 2016, the mean concentrations of glyphosate and AMPA were 0.22 mg kg^−1^ and 0.31 mg kg^−1^, respectively.

### 3.3. Air

The spread of herbicides in the air is one of the main pathways for these chemicals to enter the environment [82]. Herbicides in the air are the result of drift associated with their application, and are subject to evaporation and displacement by fine spray particles; drift of vapors after they have been applied to an area; and drift with wind or wind-borne soil particles. Air pollution from herbicides also depends on the extent of the areas where the spray has been applied [83]. Of great importance during the movement of herbicides in the air is droplet size, which determines the competition between herbicide absorption by plant surfaces and oxidation. Droplets of small size tend to evaporate much more rapidly than droplets of larger size, which in turn are absorbed more quickly [84].

As reported by Bish et al. [85], some of the active ingredients in herbicide formulations are volatile and can evaporate depending on the substance’s vapour pressure in the atmosphere, temperature and relative humidity. Evaporation of herbicides can be reduced by precipitation, with precipitation causing increased leaching [86].

The ability of herbicides to volatilize and enter the atmosphere as a gas depends on several of their properties, i.e., vapor pressure, solubility in water and adsorption coefficient. However, the most important characteristic is vapor pressure, since herbicides can freely transform from a liquid or solid state to a volatile state. Herbicides with higher vapor pressure are more likely to volatilize into the atmosphere [42]. The volatilization of herbicides is as important in the transfer of compounds from the soil or plant surface to the atmospheric air as the chemical or microbial degradation of herbicides. The volatilization of herbicides from the plant surface is much faster than from the soil surface. This process can occur on the plant surface up to 3-fold faster than on the soil surface. When herbicides volatilize, they can be transported horizontally over considerable distances. If the compounds remain in the lower troposphere, then the movement of herbicides is regulated by the climatic conditions of the area. If herbicides are rapidly displaced to the middle and upper troposphere, then their residence time increases with range [87].

Boonupara et al. [84] report that many groups of pesticides, including herbicides, have been detected in ambient air, and examples of herbicide active ingredients and their contents in the air are shown in Table 3.

A study by Zaller et al. [83] in an area of eastern Austria showed that, of all the pesticides detected in the air, up to 36.0% were herbicides. The most frequently detected herbicide active ingredients were metolachlor, terbuthylazine, pendimethalin, prosulfocarb and 2.4-D-ethylhexyl. The frequency of their occurrence in air samples ranged from 93.3% to 100%. In terms of toxicity, 50.0% of the active substances were classified as eye irritants, 46.0% as acute toxics or skin irritants, 42.0% as skin sensitizers, 33.0% as toxic to the reproductive system, 21.0% as toxic to a specific organism, 17% as carcinogens and 4.0% as endocrine disruptors. Morton et al. [88] report that about 0.3% of MCPA can volatilize from soil into the ambient air. Although this is a small amount, MCPA was detected in 50.0% of the samples in the air taken in Canada, with concentrations ranging from 1.9 ng m^−3^ to above. Within Canada, two herbicide-ingredient substances 2.4-D and MCPA, which belong to the phenoxyacid group of compounds, were detected in the ambient air in agricultural prairie regions. The content of these substances ranged from 0.11 to 2.73 ng m^−3^, and 0.17 to 1.88 ng m^−3^, respectively [89].

## 4. Effects of Herbicides on Organisms Other than Weeds

All over the world, a reduction in the biodiversity of organisms is observed in the environment, through a reduction in populations of, for example, insects, earthworms and birds [90,91,92,93]. Such disturbances in ecosystems are the result of stress factors, among which herbicide use is a huge contributor [94]. These chemicals are not only a threat to plants and animals, but also pose serious risks to human health and life [95,96].

### 4.1. Humans and Animals

The common use of herbicides worldwide has led to environmental contamination, which entails increasing concerns over their bioaccumulation and related chronic risks posed to human and animal health. Although herbicides serve for weed control, they may directly or indirectly affect other organisms also [97,98]. Their reported multiple side-effects on non-target organisms include, e.g., impeded navigation and larvae development in honeybees. Honeybees may be exposed to herbicides via immediate contact with plant protection agents or via oral contact with contaminated pollen or nectar [99,100,101,102]. In addition, their contact with herbicides is likely to result from leaf spraying, drift and soil contamination [103,104,105]. In a study conducted by Hladik et al. [106] on grassy areas and arable fields in north-eastern Colorado, 19 pesticides were detected in dead wild bees, including insecticides, fungicides and herbicides. Of the latter group of chemicals, atrazine and metolachlor accounted for 19.0% and 9.0%, respectively. Hoopman et al. [107], who analyzed the effect of glyphosate on honeybee drone sperm, observed an increased number of spermatozoa along with an increasing glyphosate dose, where LD_50_ was at 0.31 mg cm^−3^. In turn, Migdał et al. [108] noted that the consumption of sugar syrup containing bentazone and metamitron by honeybees contributed to their increased aggression and mortality within 168 h post-consumption. In the case of honeybees, herbicides also lead to the impairment of their sensorial capabilities. For instance, Mengoni Goñalons and Farina [109] demonstrated suppressed response to saccharose and impaired sense of smell in honeybees after their long-term exposure to glyphosate. Such responses of these insects to herbicides were also confirmed by Dai et al. [101] and Vázquez et al. [110]. In turn, lethal doses (LD_50_) of ametrine, atrazine and clomazone to aquatic insects (*Limnocoris submontandoni*) reached 1012.41; 192.42 and 46.09 mg dm^−3^ [111], respectively.

Earthworms represent another group of organisms particularly exposed to herbicides via absorption of these chemicals dissolved in soil and water, through the skin, and via ingestion of contaminated soil, plants or organic matter [112]. For example, glyphosate was reported to cause their reproduction rate and biomass to decrease, and to cause DNA damage [113,114]. Other herbicides detected in earthworm samples from agricultural areas included pendimethalin, diflufenican and pyroxsulam, according to Pelosi et al. [115].

Birds are also exposed to the adverse effects of herbicides, mainly through the ingestion of contaminated seeds and small insects or water, as well as by inhalation, absorption through the skin and feather preening [116]. According to Katagi and Fujisawa [69], when pesticides (including herbicides) pervade a bird’s body, they may bioaccumulate in its liver, muscles, fat, and eggs. An example in this case is glyphosate, which may lead to brain and lipid damage in birds and to embryo development disorders in eggs [117]. The bodies of birds are capable of metabolizing these chemicals in two phases, the first of which involves the processes of oxidation, reduction and bond cleavage, whereas the second one entails conjugation. The microsomal fraction contains enzymes of the P450 cytochrome, which act as monooxygenases, and uridine-diphosphate glucuronic glucuronosyltransferase. The enzymes mediating herbicide metabolism, i.e., glutathione-*s*-transferase and glutathione sulfotransferase, are located in the cytosolic fraction, whereas esterases are found in the microsomal and cytosolic fractions [69].

Aquatic animals absorb herbicides most commonly through the gills, skin and intestinal epithelium. When herbicides reach the bloodstream, they further migrate to various tissues and may trigger various disorders in cell permeability, ion and electron transport, and activities of enzymes associated with cell membrane. They may also affect functions of cell organelles, leading to the induction of cell apoptosis and necrosis or to the activation of cell tumorigenesis [118]. However, these chemicals may be excreted by aquatic animals with urine and feces, but they may also reach their liver through the circulatory system, where they are metabolized by detoxifying enzymes secreted in hepatocytes. Furthermore, when combined with antioxidants (e.g., glutathione), they are rapidly excreted from the body. Reactive oxygen species and secondary metabolites formed during herbicide degradation remain in animal bodies and become toxic to them by inducing oxidative stress [118,119]. Some phenyl-urea herbicides, e.g., diuron and linuron, act in the environment as antagonists to androgen receptors in fish and mammals and may adversely affect mitochondrial bioenergetics and ATP generation [120]. Marlatt and Martyniuk [97] reported LC_50_ doses of linuron ranging from 3.2 to 16.4 mg dm^−3^ for *Oncorhynchus mykiss*, and to reach 2.9, 5.2 and 16.2 mg dm^−3^ for *Ictalurus punctatus*, *I. nebulosus* and *Lepomis macrochirus*, respectively. In addition, linuron was reported to disrupt thyroid functions in mammals, fish and amphibians. In turn, Hogan et al. [120] demonstrated its toxic effects on tissues and on the puberty and reproductive period in rats. A study conducted by Coullery et al. [121] showed that glyphosate applied at a dose of 4000 mg dm^−3^ impaired the development of nerve cells and the growth of axons in rats. According to Matozza et al. [122], aminomethyl-phosphonic acid, being a metabolite of glyphosate, may adversely affect cells and biochemical processes occurring in mussels (*Mytilus galloprovincialis*). Long-term exposure to glyphosate and 2,4-D was also found to lead to growth inhibition and suppression of swimming activity in tadpoles belonging to the species *Boama faber* and *Leptodactylus latranas*. What is more, these organisms manifested disorders in the development of erythrocytes and damage to the gastrointestinal tract, intestines in particular [123]. Similar observations were made in the research by Vurm et al. [124], who demonstrated that Roundup Classic Pro (glyphosate—28.85%, and ether alkylamine ethoxylate—6%), Kaput Premium (glyphosate—28.85%, N-(phosphor-methyl)glycine—41.5%, and amine salt of phosphate ester—from 5.0% to 15.0%), Banvel 480 S (3,6-dichloro-o-anisic acid + dimethylamine—from 30.0% to 50.0%), Lontrel 300 (clopyralid monoethano-lamine salt—35.0%, and alkylphenol alkoxylate—5.0%), and Finalsan (nonanoic acid—18.67%, and propan-2-ol—4.0%) herbicides were toxic to *Artemia salina* crustaceans, with the strongest adverse impact noted for Roundup Classic Pro. The toxicity (LD_50_) of selected herbicides to honeybees, earthworms and birds [96] is presented in Table 4.

By contaminating various ecosystems, herbicides enter the food chain and pose a severe threat to human health. Their impact on human health is determined by their type, dose, penetration route, and exposure time [23]. Ample studies [128,129,130,131,132,133,134,135,136] have proved that herbicides exert this strong effect on human health as they act as neurotoxic, endocrine, carcinogenic, metabolic, and developmental factors.

Herbicides are absorbed by humans through drinking water contaminated with these chemicals. This pertains especially to glyphosate and metolachlor [116]. Herbicides may also enter the human body also through the inhalation of contaminated air, which is a common case among persons working on farms [137,138]. These chemicals cause damage to the placenta during pregnancy, inhibit aromatase, and cause fluctuations in the amount of mRNA [116,139]. They can also trigger changes in the number of red blood cells by increasing the level of methemoglobin. Furthermore, they can lead to cardiac arrhythmia, hypertension, kidney and respiratory failure, and even mental illness in humans [140]. Glyphosate has been found to be particularly toxic to humans, as it can cause damage to cell membranes and DNA, impede mitochondrial functions and cause cancers [141]. Persons exposed to herbicides (glyphosate) have been reported to suffer also from kidney failure; mental illness, autism, ADHD, Alzheimer’s diseases or Parkinson’s disease [142,143]; dermatological diseases; and respiratory tract diseases [144]. In another study conducted by Kwiatkowska et al. [145], exposure to glyphosate doses from 85.0 to 1690 mg dm^−3^ of peripheral blood cells has led to DNA damage in leukocytes. In turn, a glyphosate dose of 42 mg dm^−3^ suppressed DNA methylation, which might have upset the balance between cancer cell proliferation and apoptosis. Such disorders may further result in the activation of oncogenes [146]. Another strongly toxic herbicide is paraquat, which poses severe threat to human health as it induces vomiting, diarrhea, loss of electrolytes and fluids, and even leads to death when present in the human body in an amount of 30 mg kg^−1^ body mass [147]. Acute exposure to paraquat as a result of its ingestion, inhalation or contact with the skin may be the cause of damage to many human organs, i.e., heart, liver, kidneys, spleen, adrenal glands, muscles, and the nervous system, and ultimately lead to death, which may occur within 24 h. Lung, stomach and gallbladder cancers have also been reported in persons severely exposed to paraquat, which was associated with its accumulation in their internal organs [148]. Herbicides belonging to the group of phenoxylic acids, e.g., 2,4-D and MCPA, can cause abdominal pain and headaches, vomiting, diarrhea, hypertension and even gastrointestinal bleeding. Moreover, they cause irritation and itching of this skin, and their penetration through the respiratory tract impairs breathing [149]. Arici et al. [150] have emphasized a highly toxic effect of another herbicide, pendimethalin (a compound from the dinitroaniline class), on the human body. Persons who have come into contact with this herbicide have experienced damage to the digestive tract, manifested as vomiting and epigastric pain. In addition, too long exposure to pendimethalin has been reported to increase the risk of development of lung, pancreatic and rectal cancers.

### 4.2. Plants

Weeds strongly affect plant production in the agricultural sector as they may hamper crop growth and development through, i.e., competition for nutrients or access to sunlight. One of the solutions to this problem is the use of herbicides, which eradicate weeds quickly and effectively [151,152]. Herbicides still remain the main tool for weed control because they are highly effective and convenient. Most herbicides used in agriculture have been designed to act on weeds at various points of the photosynthesis pathway, including by hindering energy production and electron transport, and blocking ATP production. An example in this case is that of phenyl-urea herbicides, which inhibit the photosynthesis process in photosystem II and can affect other proteins in the electron transport chain additively or synergistically, associated with plant growth and development [97]. Herbicides, by inhibiting specific enzymes in weeds, i.e., coenzyme A carboxylase, acetolactate synthase, and 5-enolpyruvylshikimate-3-phosphate synthase, cause disturbances in fatty acid and amino acid synthesis. Following the application of these chemicals, weed growth can stop within hours, causing shoot and root dwarfing, leaf yellowing and death within days or weeks [18]. A study by Ziveh and Mahdavi [153] showed that the herbicides tested (sulfosulfuron, mesosulfuron + idosulfuron, metsulfuron methyl + sulfosulfuron, isoproton + diflufenican, clodinafob-propargyl, pinoxaden, diclofop-methyl, pinoxaden + clodinafob-propargyl, fenoxaprop-p-ethyl + mefen-pyper-d-ethyl, and flam-prop-m-isopropyl) significantly reduced the total density of narrowleaf weeds (from 50% to 100%). The exception was the herbicide tralkoxidim, which inhibited weed growth by only 7%. The herbicides analyzed had no negative effect on the growth and development of the crop *Triticum rimpaui* Wittm.

Qi et al. [154] report that atrazine and tribenuron-methyl applied to fallow land in China’s Huang-Huai-Hai Plain near Beijing significantly altered plant species composition, reduced the number of plant species and relative frequencies of some plants, and significantly reduced the Margalef species richness index and Shannon diversity index of the plant community. Atrazine binds to the plastoquinone binding site in the photosynthetic electron transport system and inhibits photosynthesis, while tribenuron-methyl inhibits acetolactate synthase, which participates in the biosynthesis of branched-chain amino acids. Such effects of herbicides may have contributed to changes in wild plant communities.

Successive use of herbicides with similar modes of action can contribute to the development of weed resistance. There is always the possibility that weed species that have developed resistance to a particular herbicide may become resistant to other herbicides with similar modes of action. In addition, weed resistance to herbicides can exacerbate yield losses. To date, as many as 523 cases of unique resistant weeds have been reported worldwide, which are resistant to 167 herbicides used in 99 crops [155]. The effectiveness of herbicides in controlling weeds depends primarily on the weed species, the herbicide application rate and timing, and environmental conditions [156,157].

The growth and development of crops is inhibited by the accumulation of herbicides in these plants and is the cause of a number of metabolic disorders, such as changes in the mechanism of the photosynthetic electron transport chain and reduction in the total content of chlorophyll *a* and *b* and carotenoids [158]. The use of herbicides from the group of organophosphate compounds, e.g., glyphosate, blocks the synthesis of aromatic amino acids (phenylalanine, tryptophan, and tyrosine) by affecting the EPSPS (5-enolpyruvate-shikimate-3-phosphate synthase enzyme) of the shikimic acid pathway. For example, in cotton plantations, glyphosate can affect the distribution of seed capsules of this plant, which is the cause of reduced yield, fiber quality and percentage of ginning. It also diminishes reproductive performance of plants by triggering changes in flower morphology, pollen viability and pollination efficiency [159]. A sub-toxic concentration of glyphosate may also adversely affect soybean plants, including traits of their root secretions, like enhanced secretion of amino acids by plant roots [160]. Glyphosate application may also impair the uptake of microelements by plants and their further transport, thereby inhibiting plant growth and diminishing resistance to pathogens [161]. Another herbicide often found in the environment is clopyralid (3,6-dichloro-2-pyridinecyboxylic acid), whose residues were detected in crops (cereals in particular) and also in plant products, i.e., wheat and barley bran and compost [162,163]. In turn, Baćmaga et al. [164] in our previous study of Alister Grande 190 OD herbicide containing a mixture of three active substances (diflufenican, mesosulfuron-methyl and iodosulfuron-methyl-sodium), showed its adverse effect on spring wheat development when applied to the soil in doses of 18.240 and 36.480 mg kg^−1^, which became lethal to plants. In turn, an experiment conducted by Tomkiel et al. [165] demonstrated that a mixture of active substances: flufenaced + isoxaflutole inhibited the growth and development of maize when applied in doses ranging from 5.0 to 160 mg kg^−1^. This preparation was also observed to cause damage to maize seedlings as early as in the germination stage. A very high sensitivity of maize to another herbicide, Successor T 550 E (terbuthylazine and pethoxamide),was found by Wyszkowska et al. [166]. The resistance index of maize reached 0.75 to a Successor T 550 E dose of 0.73 mg kg^−1^ and merely 0.05 to a dose of 14.63 mg kg^−1^.

Most crops can absorb herbicides from the natural environment. The exposure of plants to herbicides results in the generation of reactive oxygen species, an increased number of antioxidative enzymes in plants, and expression of genes responsible for herbicide degradation. A typical antioxidant enzyme secreted by plants is peroxidase, which catalyzes the removal of H_2_O_2_ by oxidizing a specific chemical substance, e.g., herbicide metabolites or phenols. Its activity mitigates plant cell damage caused by reactive oxygen species [4]. Herbicides may also be degraded via metabolic processes occurring in plants. Their conversion is mediated by glutathione-s-transferase, which leads to the conjugation between a herbicide and a tripeptide [134]. During oxidative stress, excessive amounts of reactive oxygen also activate signaling molecules in a plant. One such signaling molecule is salicylic acid, which not only protects the plant against pathogens but is also involved in herbicide degradation in the plant [50,167,168].

Herbicides adversely affect not only terrestrial plants but also aquatic plants, e.g., *Lemna minor*, whose exposure to glyphosate and its metabolite aminomethyl (phosphonic) acid (AMPA) caused morphological changes in plants, with glyphosate being 1.5 times more toxic than its metabolite [169]. Herbicides from the group of triazine compounds (simazine, atrazine, and metribuzin) and phenyl-urea class compounds (linuron, diuron, and isoproturon) have been shown to block the electron transfer in the PSII photosystem in the course of photosynthesis. In turn, isoproturon has been reported to suppress carbon fixation and oxygen production [170], whereas, herbicides impeding hormonal processes, i.e., picloram, clopyralid, triclopyr, 2,4-D and 2,4,5-T [171] were considered particularly dangerous to vascular plants.

Aquatic plants are capable of neutralizing herbicides present in water and bottom deposits via specialized mechanisms. Damaged aquatic plants may absorb herbicides by both the root system and leaves. However, the rate of herbicide absorption by the root and the shoot differs [172], being very rapid through the roots, e.g., the absorption of phenyl-urea herbicides by *Lagarosiphon major* occurs within 24 h. Herbicide uptake may also proceed via the transport across the cell membrane owing to the specific binding with D1 protein of the PSII photosystem complex, e.g., isoproturon and chlorotoluron [124]. Heine et al. [173] have reported that herbicide translocation in the plant occurs via the phloem or xylem, e.g., the uptake of linuron by the shoots of *Elodea canadensis* and *Myriophyllum spicatum*, in which the translocation of this herbicide occurred within 3 days [174].

### 4.3. Microorganisms

Weed control practices trigger major changes in the population and diversity of microorganisms, the rhizosphere, and thus in the physical and chemical properties of the soil. Herbicides affect soil microorganisms, their community structure, and ecological functions, with most of these effects being minor and transient [175]. Herbicides affect microorganisms by inducing changes in their abundance, enzymatic activity, cell membranes, and cell wall composition [74,176].

An adverse effect of herbicides on microorganisms has been described in a study by Jabłońska-Trypuć et al. [177], who demonstrated cytotoxic effects of bifenox and dichlobenil on *Escherichia coli* bacteria and *Candida albicans* yeast. In the case of *Pseudomonas fluorescens*, cell viability was observed to increase significantly, regardless of herbicide dose. In turn, a study by Medo et al. [178], conducted with linuron and dimethachlor herbicides, showed changes in the structure and activity of microbial communities, including reduced population numbers of Proteobacteriota and *Nitrosospirae* bacteria, and increased population numbers of Gemmatimonadetes and Saccharibacteria after linuron treatment. In turn, the application of dimethachlor caused a significant increase in the population number of the Proteobacteriota phylum bacteria and a decrease in the number of Acidobacteriota. Investigations conducted by Łozowicka et al. [179] revealed a cytotoxic effect of sulfosulfuron on *P. fluorescens* bacteria. In turn, the application of MCPA herbicide was found to stimulate *B. cereus* and *P. fluorescens* bacteria. A mixture of terbuthylazine, mesotrione and s-metolachlor may be harmful to the soil environment, which was confirmed by Borowik et al. [180]. A mixture of these active substances introduced into the soil in doses ranging from 0.67 to 430 mg kg^−1^ led to changes in the structure of organotrophic bacteria, actinobacteria, and fungi, as well as in fungi biodiversity. In turn, Kucharski et al. [181], who evaluated the effect of Boreal 58 WG herbicide (flufenaced and isoxaflutole), did not observe any significant changes in the proliferation of organotrophic bacteria, actinobacteria and fungi after soil treatment with herbicide doses ranging from 0.25 to 40 mg kg^−1^.

The use of herbicides, the orthophosphate class in particular, poses a potential threat to microorganisms, whereas their toxicity is determined by exposure time. The 2,4-D herbicide may reduce population numbers of the *Rhizobium* genus bacteria and suppress activities of phosphatase and nitrogenase [15]. Glyphosate affects soil microorganisms, the composition of their communities and their ecological functions, but most of these effects are minor and transient [182]. When applied at recommended or lower doses, it negatively affects the proliferation of microorganisms promoting plant growth and development, including *Burkholderia* sp., *Pseudomonas* sp., *Rhizobium* sp., and arbuscular mycorrhiza [57,183]. Newman et al. [184] observed that the long-term use of this herbicide modified the structure of Acidobacteriota and *Xanthamonadales* bacterial communities by increasing their proliferation and impeding their carbon metabolism. The adverse effects of metazachlor on the population of soil microorganisms were reported by Baćmaga et al. [185]. Doses ranging from 6.666 to 213.312 mg kg^−1^ applied to the soil inhibited the proliferation of oligotrophic bacteria and their endospore forms, organotrophic bacteria, *Azotobacter* sp., actinobacteria, and fungi. Its effects were also tangible in the colony development index (CD) and eco-physiological diversity index (EP) of microorganisms, the values of which decreased significantly. A mixture of pethoxamide and terbuthylazine may upset biological homeostasis in the soil, as confirmed in the study by Wyszkowska et al. [186]. Their mixture inhibited the proliferation of the *Azotobacter* genus bacteria, spore-forming oligotrophs, actinobacteria and fungi.

Herbicides may also exert positive effects 0n soil microbiota, as shown in the study by Tomkiel et al. [187], who analyzed outcomes of carfentrazone-ethyl application. When used in doses ranging from 0.264 to 168.96 µg kg^−1^, it increased the population numbers of organotrophic bacteria, *Azotobacter* genus bacteria, actinobacteria, and fungi on day 60 as well as spore-forming organotrophic bacteria on day 30.

In turn, Baćmaga et al. [188] demonstrated that herbicide preparations: Sulcogan 300 SC (sulcotrione), Tezosar 500 SC (terbuthylazine), and Sulcotrek 500 SC (sulcotrione + terbuthylazine mixture), applied to the soil in doses of 1.50 mg kg^−1^, 1.65 mg kg^−1^ and 3.33 mg kg^−1^, respectively, increased the counts of bacteria from the Actinobacteriota and Proteobacteriota phyla that were most heavily populating the soil tested. Fungi showed various responses to these herbicides, namely counts of the fungi from the Ascomycota and Mortierellomycota phyla decreased after soil exposure to Sulcogan 300 SC and increased after soil treatment with Sulcotrek 500 SC, whereas all herbicides tested exerted an inhibiting effect on the fungi from the phylum Basidiomycota. Another research by Baćmaga et al. [189], aimed to evaluate the effect of active substances of sulcotrione and terbuthylazine on soil microorganisms, also demonstrated changes in the structure of bacterial and fungal communities. Sulcotrione applied at a dose of 1.15 mg kg^−1^ and terbuthylazine at a dose of 1.65 mg kg^−1^ reduced the OTU number of Actinobacteriota bacteria and increased that of Proteobacteriota. The Ascomycota fungi prevailing in the soil practically did not respond to the analyzed active substances.

Adverse effects of herbicides have also been reported in respect of aquatic microorganisms, e.g., microalgae, bacteria and protozoa, and are manifested by changes in, e.g., synthesis of amino acids, chlorophyll production, course of photosynthesis, and respiration. For instance, *Vibrio fischeri* bacteria and microalgae were sensitive to glyphosate present in water, with EC_50_ ranging from 5.4 to 7.6 mg dm^−3^ for *V. fischeri* and from 1.2 to 7.8 mg dm^−3^ for microalgae [57]. Microalgae are also sensitive to herbicides found in water, which was confirmed in the study by Rodriguez-Gil et al. [190]. Glyphosate applied at a dose of 1.2 mg dm^−3^ inhibited photosynthesis, decreased density and impeded the growth rate of microalgae cells. In turn, doses ranging from 2.7 to 2.9 mg dm^−3^ stimulated the proliferation of bacterioplankton and picocyano-bacteria and enhanced photosynthetic activity in periphytic algae, which was explained by an increase in the phosphorus content in water after glyphosate conversion [191,192]. Vurm et al. [124] evaluated toxicity of four active substances: glyphosate, dicamba, clopyralid and pelargonic acid, to the marine bacterium *Aliivibrio fischeri*. The half-maximal inhibitory concentrations (IC_50_) determined for these individual substances were 7934 µg dm^−3^; 15,937 µg dm^−3^; 10,417 µg dm^−3^; and 16,040 1 µg dm^−3^, respectively. These values permitted the conclusion that the analyzed active substances showed acute toxicity to *A. fischeri*. In turn, Muturi et al. [193] did not report any significant effect of atrazine and glyphosate applied alone and in a mixture on the structure of aquatic bacteria. The water habitat was most densely populated by bacteria belonging to the following phyla: Proteobacteriota (62.6%), Bacteroidetes (15.0%), and Firmicutes (9.07%).

**Figure 3:**
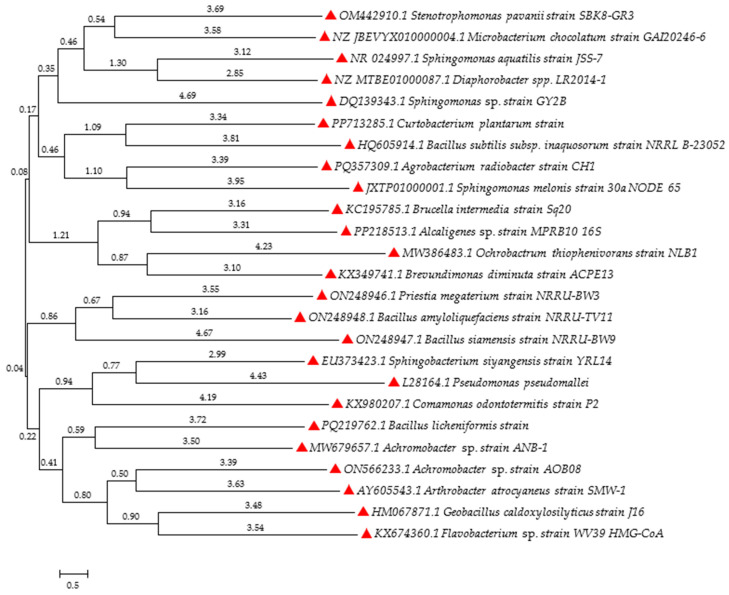
Examples of bacteria showing potential to biodegrade herbicides are presented in the form of a phylogenetic tree based on sequences found in the NCBI (National Center for Biotechnology Information) database. The phylogenetic tree was created using MEGA 7 software by the neighbor-joining method [195].

There are many methods of environmental treatment regarding herbicides, such as chemical precipitation, oxidation, reduction, filtration, ion exchange or electrochemical treatment. However, a promising alternative in this respect is offered by microorganisms, which have a huge potential for biodegradation or neutralization of these xenobiotics to less harmful compounds [194]. Examples of bacteria capable of converting herbicides are shown as a phylogenetic tree based on nucleotide sequences downloaded from the NCBI database (Figure 3).

Organophosphate herbicides can be degraded by microorganisms to less harmful compounds. The potential for herbicide degradation in contaminated soils has been demonstrated for multiple bacteria genera, including *Achromobacter*, *Alcaligenes*, *Arthrobacter*, *Bacillus*, *Microbacterium*, and *Ochrobactrum*. Strains of these bacterial genera not only degraded herbicides but also produced plant growth-stimulating regulators [196]. In addition, microorganisms are highly potent to use herbicides as sources of nutrients, including e.g., carbon, nitrogen, phosphorus, and sulfur [197].

Herbicides, in turn, may induce oxidative stress in cells of microorganisms by generating various radicals. However, certain bacteria, like *E. coli*, can overcome this stress by secreting antioxidative enzymes encoded by Mn-SOD (*sodA*) and Fe-SOD (*sodB*) genes. Manganese and iron (Mn and Fe), being constituents of antioxidative enzymes, ensure their stability, catalysis and appropriate structure. Mutations proceeding in SOD genes may modify both the metabolic and antioxidative reactions in cells of *E. coli* strains [198]. *Pantoea ananatis* possesses polymorphic catalase enzymes that control oxidative stress. Resistance of these bacteria to herbicides (mainly mesotrione) is associated with changes in lipid membrane saturation, leading to increased membrane impermeability and increased formation of glutathione-s-transferase-mesotrione conjugates, which in turn increases the level of herbicide degradation. Modifications in membrane lipid saturation in bacteria can act as selective barriers against herbicides [199].

Another example of bacteria resistant to herbicide-induced oxidative stress is *P. ananatis*, which has been isolated from agricultural soils. This bacterium has the ability to multiply in the presence of mesotrione, because its cells contain a polymorphic catalase able to control oxidative stress. In addition, it shows changes in the saturation of the lipid membrane, which leads to an increase in its impermeability and the formation of GST-mesotrione (glutathione-s-transferase- mesotrione) conjugates, which in turn increases the effectiveness of herbicide degradation [188,200].

Oxidative stress induced by herbicides can also be alleviated in bacteria by their accumulation in stress metabolites, i.e., polysaccharides, proline, abscisic acid and glycine betanin, and by regulating the synthesis of enzymatic and non-enzymatic antioxidants, e.g., catalase, antioxidative enzymes, ascorbate peroxidase, ascorbic acid, glutathione reductase, and glutathione [201].

*P. fluorescens* and *Bacillus cereus* revealed vast potential for the rapid biodegradation of herbicides, including those from the sulfonylurea and phenoxy groups. *B. cereus* degraded these herbicides within 7.7 to 8.4 days, whereas *P. fluorescens* within 10.2 to 10.5 days [72].

Glyphosate is one of the most commonly used herbicides in plant protection against weed. It can be used by many microorganisms as a source of phosphorus, whereas certain bacteria, e.g., *Arthrobacter* sp. GLP, *Streptomyces* sp. StC, and *Achromobacter* sp. LW9, use it as a source of nitrogen and carbon. Enzymatic degradation of glyphosate consists in the direct cleavage of the C–P bond, producing sarcosine and inorganic phosphorus. The bond is broken down by a multi-enzyme complex called C–P lyases, which contains components responsible for phosphate uptake by the cell, regulatory and auxiliary components, and enzymes that catalyze the cleavage of the carbon–phosphorus bond. Such glyphosate-degrading capability was demonstrated for *E. coli*, *Achromobacter* sp. MPS 12A, *Arthrobacter* sp. GLP_1, and *P. putida* bacteria [202,203]. In addition, many genes are involved in glyphosate degradation, depending on the intermediate product formed. The formation of sarcosine as an intermediate product is mediated by genes encoding C–P lyase clustered in the *phn* operon. In turn, the following genes, *glpA* (gena gene homologous with genes of hygromycin phosphotransferase) and *glpB*, are involved in herbicide degradation resulting in the formation of aminomethyl (phosphonic) acid (AMPA) as an intermediate product. Other genes associated with glyphosate degradation include the genes of oxidoreductase *gox*, which are responsible for its conversion to glyoxylate and AMPA [204].

**Figure 4:**
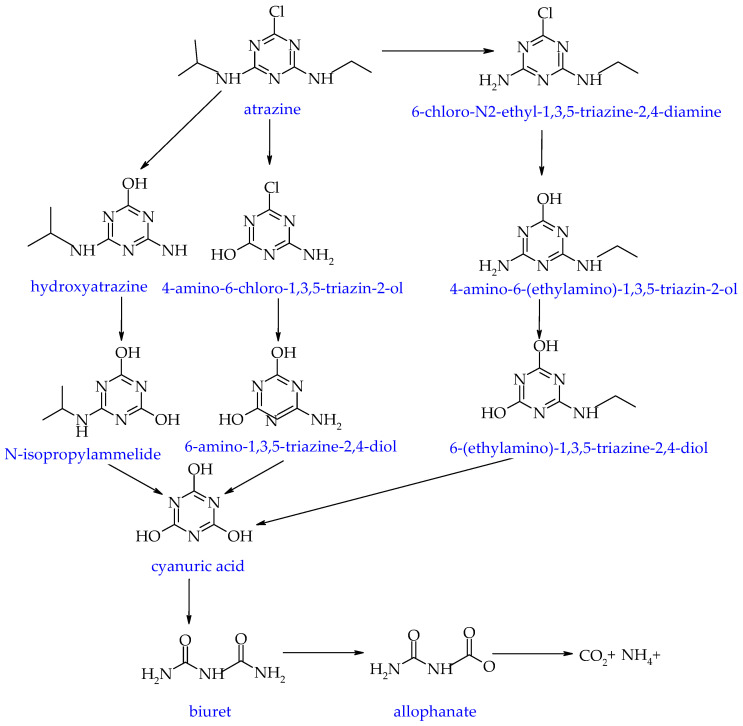
Biodegradation of atrazine in the environment by bacteria. The atrazine degradation pathway was made using ISIS Draw 2.3 software [30]. The process of biodegradation of herbicides by microorganisms is most often described on the basis of degradation pathways carried out by bacterial strains.

Another herbicide commonly applied in agriculture is atrazine. Its biodegradation proceeds via dechlorination, dealkylation, hydroxylation, and ring cleavage (Figure 4). The *atzA*, *atzB*, *atzC*, *atzD*, *atzE*, and *atzF* genes of the *Pseudomonas* sp. AKN5 bacteria strain are responsible for its complete mineralization in the dechlorination pathway. *Bacillus velezensis* MHNK degrades cyanuric acid to urea and then to carbon dioxide and ammonia via the *atzA*, *atzB* and *atzC* genes [205].

The metabolic pathway for the degradation of the herbicide dicamba involves dechlorination and demethylation of the molecule, leading to the formation of 2-chloromaleylpyruvate. The degradation of this herbicide occurs in stages. At first, the dicamba herbicide molecule, under the influence of the enzyme dicamba methyltransferase, is converted to 3,6-dichlorosalicylate, and under the influence of the enzyme 3,6-DCSA 5-hydroxylase to 3,6-dichlorogentisate. In the next step, 3,6-dichlorogentisate is transformed to 3-chlorogentisate by the action of 3,6-DSCA 6-dehalogenases, and then to 2-chloromaleylpyruvate by the action of the enzymes 3-chlorogentisate 1,2-dioxygenase or gentisate 1,2-dioxygenase. The ability to transform the herbicide dicamba is demonstrated by the bacterial strain *Sphingomonas* sp. RW5 [206].

The ability to biodegrade 2,4-dichlorophenoxyacetic acid (2,4-D) is demonstrated by the *Cupriavidus necator* bacterial strain JMP134. The enzymes produced by these bacteria act in stages, leading to the breakdown of harmful halogenated aromatic compounds into harmless secondary metabolites. These bacteria possess the *tfdA* gene encoding an α-ketoglutarate-dependent 2,4-D dioxygenase, which catalyzes the cleavage of the ether bond in 2,4-D and converts it to 2,4-dichlorophenol (2,4-DCP). The 2,4-DCP hydroxylase encoded by *tfdB* then hydroxylates the phenolic compound, resulting in the formation of 3,5-dichlorocatechol (3,5-DCC). The 3,5-DCC undergoes ortho- or meta-cleavage under the influence of chlorocatechol 1,2-dioxygenase encoded by *tfdC*, resulting in the formation of 2,4-dichloro-cis, cis-muconate (DCMA). The resulting compound is further converted to cis-2-dichlorodienolactone (CDL) by the action of chlorocatechol cycloisomerase encoded by *tfdD*, and then to 2-chloromaleylacetate (CMA) by the action of chlorodienolactone hydrolase encoded by *tfdE*. Further, 2-chloromaleylacetate (CMA) is reduced to maleylacetate by chloromaleylacetate reductase. The final step produces 3-oxoadepane, which enters the tricarboxylic acid cycle through *tfdF* encoded maleylacetate reductase [207]. The catabolic pathway of the herbicide lactofen occurs with the participation of *Brevundimonas* sp. LY-2 consists of the hydrolysis of benzene alkyl esters, during which the nitrile group on the aromatic ring is reduced to an amino group. Subsequently, acetylation and dechlorination occur simultaneously on the phenyl ring, after which the carboxyl and acetyl groups are reduced to hydroxyl groups [208].

Another example is the biodegradation of the herbicide ioxynil octanoate carried out by two strains of bacteria *Lysinibacillus boronitolerans* MLH-31 and *Bacillus cereus* MLH-61. During the transformation of this compound, four metabolites are formed: 3,5-diiodo-4-hydroxybenzamide, 3,5-diiodo-4-hydroxybenzoic acid, the mono-deiodinate compound 3-iodo-4-hydroxybenzoic acid and the product of the cleaved Caromatic-CN 1,3-diiodophenol. The transformation of ioxynil octanoate probably involves enzymatic systems, evidencing the presence of esterases, nitrile hydratases, amidases, nitrilases, dehalogenases and lyases [209].

**Figure 5:**
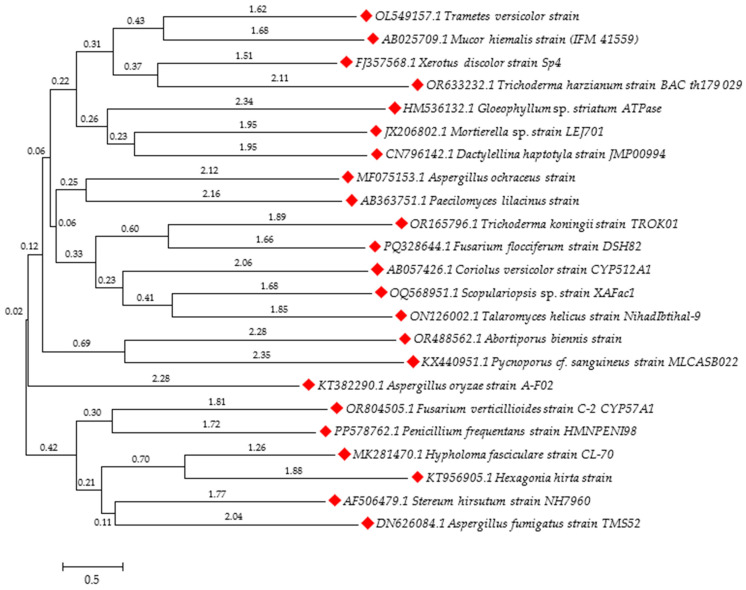
Examples of fungi showing potential to biodegrade herbicides are presented in the form of a phylogenetic tree based on sequences found in the NCBI (National Center for Biotechnology Information) database. The phylogenetic tree was created using MEGA 7 software by the neighbor-joining method [195].

The process of herbicide biodegradation by microorganisms is most often described based on degradation pathways carried out by bacterial strains. However, there are many fungal strains that can degrade and metabolize herbicides (Figure 5) [210]. Examples of fungi showing herbicide degradation capabilities are presented in the form of a phylogenetic tree, based on nucleotide sequences from the NCBI database.

Fungi possess multiple enzymes, metabolic pathways and mechanisms that can be successfully used in the conversion of these organic pollutants. Most of all, they are genetically stable and quickly adapt to changing environmental conditions, including pH, temperature, redox potential and the presence of various chemical pollutants. The enzymatic mechanism of fungi includes intracellular and extracellular enzymes, i.e., laccases, peroxidases, transferases, esterases, dioxygenases, hydrolases, dehalogenases, and cytochrome P450 monooxygenases [211,212].

Fungi can degrade herbicides non-enzymatically through bioaccumulation, and enzymatically through bioconversion and biodegradation [183]. Bioaccumulation of herbicides by fungal cells occurs as a result of their sequestration through the synthesis of intracellular chelates and their dilution inside fungal cells. This process involves the transfer of these contaminants from the internal biomass of fungal cells to organelles [213]. However, the main mechanisms deployed by fungi to neutralize herbicides entail biodegradation and bioconversion. When released into the environment as a result of these processes, these compounds possess a different molecular structure and show increased solubility, which suppresses their bioactivity and makes them more susceptible to degradation [192,214].

Fungi can also degrade herbicides through metabolic processes involving enzymes, which leads to the complete mineralization of these xenobiotics. By metabolizing herbicides, fungi use them as a source of energy to satisfy their vital needs. Some species of fungi can also degrade herbicides through the synthesis of biosurfactants, which leads to the dispersion of these chemical compounds and a decrease in their surface tension, and this in turn contributes to their degradation [215].

An example of herbicide biodegradation by mold fungi is the degradation pathway of *p*-chlorophenoxyacetic acid (2,4-D) by *A. niger* Mulder, C.M.I. 31283 strain, resulting in the formation of the main metabolite, i.e., 2-chlorophenoxyacetic acid (2-CPA), followed by the hydroxylation reaction, resulting in the formation of 2-chloro-4-hydroxyphenylacetic acid and 2-hydroxyphenylacetic acid [197].

Chen et al. [207] report that the degradation of 2,4-D by *Aspergillus niger* fungi involves a stepwise transformation. First, dechlorination occurs which leads to the formation of 2-chlorophenoxyacetic acid (2-CPA), followed by a hydroxylation reaction that produces 2-chloro-4-hydroxyphenylacetic acid and 2-hydroxyphenylacetic acid. During the degradation of 2,4-D, there is also the 2,4-DCP pathway, during which catechol is formed.

The fungi *Phanerochaete chrysosporium* and *Ganoderma lucidum* are able to degrade the herbicide bentazone through the production of the enzymes lignin peroxidase, manganese peroxidase or laccase. Bentazone under the action of these enzymes is degraded by hydrolysis and photolysis to N-methylbentazone, 6-hydroxybentazone and 8-hydroxybentazone [208].

Huang et al. [209] report that only *Fusarium solani* has the potential to transform the herbicide oxadiazon. The transformation of this compound produces four metabolites: 1-(2,4-dichloro-5-isopropoxyphenyl)-1-(methoxycarbonyl)-1,2-trimethylacetyl hydrazine, l-trimethylacetyl-2-(2,4-dichloro-5-isopropoxyphenyl) hydrazine and 2,4-(dichloroisopropoxy)benzene.

Castrejón-Godínez et al. [216] indicate that there are few fungal genera with the ability to degrade glyphosate and use it as a source of nitrogen, carbon and phosphorus. These genera include *Aspergillus* sp., *Fusarium* sp., *Trichoderma* sp. and *Penicillium* sp. Biodegradation by these microorganisms can proceed with the formation of both AMPA and sarcosin. The most effective biodegradation of glyphosate is carried out by *Aspergillus oryzae* A-F02, *Trichoderma harzianum* MT871998, *Trichoderma gamsii* P2-18, *Aspergillus fumigatus* FJAT-31052, *Penicillium simplicissimum* SNB-VECD11G, *Aspergillus niger* APBSDSF96, *Aspergillus flavus* JN-YG-3-5 and *Aspergillus niger* MT871999.

## 5. Conclusions

The use of herbicides in the protection of crops against weeds is inevitable. However, despite their necessity, their selection and dosage should be carefully adjusted, not only to the weed species, but also to the species and developmental stage of the protected plant. Prudent use of herbicides can reduce the negative effects of their impact on the natural environment. Research into the development and technology of herbicides should also be increased, ensuring that plants are protected from weeds, following the principles of ecology. To ensure the proper use of herbicides in agriculture, it would be appropriate to replace synthetic herbicides with biological ones that do not have such adverse effects on the environment. A very good measure to ensure environmental safety is the continuous monitoring of ecosystems exposed to herbicides. If they become contaminated, measures should be taken to ensure that ecosystems return to biological balance. A constant and difficult challenge is to design an effective herbicide that will be effective in protecting crops on the one hand and reducing concerns about adverse effects on the environment and human health on the other. To eliminate a broad spectrum of weeds, the selection of herbicides, doses and timing of application should be tailored to the degree of weed infestation.

## Figures and Tables

**Table 1:** The most commonly used chemical groups of herbicides in crop protection.

Chemical Groupe	Active Substance	Structural Formula of the Active Substance	Crops	References
Sulfonylurea	chlorsulfuron	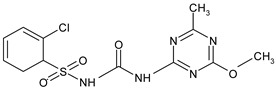	wheat, maize, rice	[23,24]
Triazine	atrazine	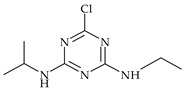	rape, wheat, maize, potatoes, beans	[25,26]
Organophosphorus	glyphosate	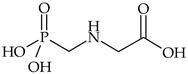	maize, wheat, soybean, cotton	[27,28]
Urea	diuron	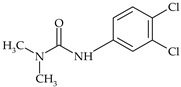	wheat, alfalfa, sugar cane, cotton	[23]
Chloroacetamide	s-metolachlor	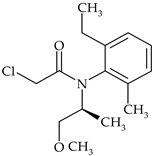	rape, maize, peas, soybean, rice	[23]
Dinitroaniline	pendimethalin	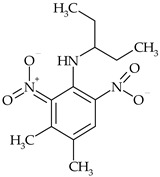	wheat, rape, peas, cabbage, carrots, rice	[26,27]
Benzoic acid	dicamba	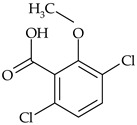	cereals, maize, soybean	[23,28]
Phenoxy acid	2,4-dichlorophenoxyacetic acid (2,4-D)	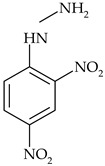	wheat, linseed, rice	[23,26]
Bipyridiliums	paraquat	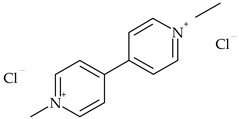	rape, pulses, potatoes	[29]
Pyrazole	pyroxasulfone	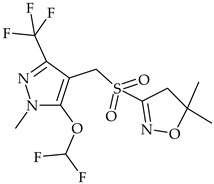	maize, soybean, field pea, sunflower	[30]
Thiocarbamate	prosulfocarb	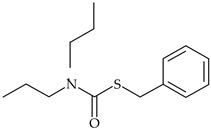	barley, wheat, potatoes	[31,32]

The structural formulas of the active substances were made using ISIS Draw 2.3 software [33].

**Table 2:** Classification of herbicides according to toxicity and hazard by oral and dermal contact using rats according to WHO.

WHO Type	Levels Toxicity	LD_50_ for the Rat [mg kg^−1^ Body Weight]	
		Oral	Dermal
Ia	Extremely hazardous	<5	<50
Ib	Highly hazardous	5–50	50–200
II	Moderately hazardous	5–2000	200–2000
III	Slightly hazardous	>2000	>2000
IV	Unlikely to present acute	>5000	

**Table 3:** Examples of herbicide content in ambient air compiled from Boonupra et al. [84].

Active Ingredient of the Herbicide	Country	Concentration Range [ng m^−3^]
glyphosate	Malaysia	503.0–517.0
	USA	0.24–0.48
	France	0.18–1.04
	Germany	20.3–3176.8
	Italy	0.10–0.30
pendimethalin	Germany	0–3916.8
	Austria	44.9–3932.4
metolachlor	Germany	0–1273.3
	Austria	12.3–382.6
diuron	South Africa	0.12
dimethenamid	Germany	0–1556.6
terbuthylazine	Germany	0–905.9
prosulfocarb	Austria	13.7–4357.8
trifluralin	France	0.12–40.74

**Table 4:** Toxicity of selected herbicides (LD_50_) to *Apis mellifera* (honeybee), *Eisenia fetida* (earthworm) and *Serinus serinus* (bird). Own elaboration based on [125,126,127].

Active Substance	LD_50_		
	*Apis mellifera* [µg bee^−1^]	*Eisenia fetida* [mg earthworm^−1^]	*Serinus serinus* [mg bird^−1^]
acetochlor	>200	105.5	928
aclonifen	107.0	307.1	13.7
alachlor	16	386.8	1536
atrazine	>100	79	4237
bentazone	>200	>1000	1140
bromoxynil	150	45	217
butachlor	>100	0.515	>4640
carfentrazone-ethyl	>81	>410	>2250
chlorsulfuron	>100	>750	>5000
chlorotoluron	>200.2	>500	272
dicamba	>89.5	>1000	188
diflufenican	>100	>500	>2150
diquat	13.0	193	0.4
diuron	>101.7	>798	1104
ethofumesate	>50	134	>2000
flufenacet	>109.2	219	1608
fomesafen	50	1000	>5000
glyphosate	>100	>5600	>2000
iodosulfuron-methyl-sodium	>150	>1000	>2000
iron sulphate	100.0	7838.0	8.4
izoxaflutole	>100	>500	>2150
linuron	>97.8	>5000	314
MCPA	>200	325	377
mesosulfuron	>13	>1000	>2000
meso-trione	>100	>2000	>3776
metazachlor	>100	500	>2000
metolachlor	>110	140	>2000
nicosulfuron	>50	>1000	>2000
oxasulfuron	>200	>1000	>2250
paraquat	9.26	>1000	35
pendimethalin	100	>1000	1421
pethoxamid	>200	316	1578
prosulfocarb	>80	71.8	>2250
rimsulfuron	>100	>1000	>2250
simazine	97	1000	4640
s-metolachlor	>200	570	2510
sulcotrione	200	>1000	>1350
sulfosulfuron	>25	>848	>2250
terbuthylazine	>32	>141.7	1236
tribenuron-methyl	>98.4	>1000	>2250
trifluralin	>100	>500	>2250
triflusulfuron	>100	>1000	>2250

## Data Availability

The raw data supporting the conclusions of this article will be made available by the authors on request.
